# Preparing Therapy Dogs for Animal-Assisted Services: Governance, Welfare-Centred Preparation, and a Lifecycle Research Agenda Across Selected European and United States Frameworks

**DOI:** 10.3390/ani16162557

**Published:** 2026-08-16

**Authors:** Muhzina Shajid Pyari, Kun Guo, Niko Kargas

**Affiliations:** 1 Independent Researcher, 1117 Budapest, Hungary; muhzina.shajid@gmail.com; 2 School of Psychology, Sports Science and Wellbeing, University of Lincoln, Lincoln LN6 7TS, UK; 3 Department of Cognitive Sciences, United Arab Emirates University, Al Ain 15551, United Arab Emirates

**Keywords:** animal-assisted services, animal-assisted intervention, therapy dog, canine welfare, human–animal interaction, handler competency, certification, One Health, zoonotic risk

## Abstract

Dog-assisted services are employed in healthcare, educational, social-care, and community settings, yet systems for preparing and protecting therapy dogs remain unevenly developed. This narrative review synthesises empirical, conceptual, and governance literature on therapy dog preparation in selected European contexts and the United States. Searches were conducted across academic databases, specialist repositories, and grey-literature sources, with evidence organised thematically around governance, candidate selection, training, handler competency, welfare monitoring, health and zoonotic risk management, and career lifecycle management. The synthesis shows that therapy dog governance remains fragmented, with international guidance, voluntary certification systems, national guideline frameworks, and legally embedded models differing in scope, enforceability, welfare safeguards, handler requirements, and re-evaluation. Across preparation domains, the review identifies a need to move beyond dog-level pass–fail certification toward assessment of the human–dog team within specific intervention contexts. Reward-based and consent-oriented preparation, multimodal welfare monitoring, handler stress recognition, veterinary screening, infection control procedures, workload regulation, and retirement planning emerge as central but inconsistently standardised requirements. The review proposes a tiered lifecycle framework conceptualising therapy dog readiness as a dynamic, welfare-centred, context-dependent process from selection through active service and retirement. Future research should prioritise longitudinal welfare assessment, validated selection and handler competency tools, comparative governance evaluation, positive welfare indicators, and transparent reporting of preparation variables.

## Simple Summary

Therapy dogs are increasingly involved in healthcare, education, social care, and community programmes, but the standards applied to their selection, preparation, assessment, and protection vary widely across organisations and countries. This review examines how therapy dogs are prepared and governed in selected European contexts and the United States, with particular attention to canine welfare, handler competency, health and infection control risks, and long-term career management. The review argues that therapy dog preparation should not be treated as a one-time certification event. Instead, it should be understood as a career-long process involving careful selection, reward-based preparation, assessment of the human–dog team, monitoring during active service, workload adjustment, and retirement planning. The review proposes a tiered lifecycle framework to help researchers, certifying organisations, practitioners, and institutions develop more consistent and welfare-centred standards. Strengthening preparation standards is important not only for the wellbeing of therapy dogs but also for human safety, public trust, and the long-term sustainability of animal-assisted services.

## 1. Introduction

Animal-assisted interventions (AAIs) comprise structured practices that intentionally incorporate animals to support therapeutic, educational, or wellbeing outcomes in human participants. Under this umbrella, animal-assisted therapy, animal-assisted education, and animal-assisted activities differ in professional oversight, goal setting, and evaluation requirements [1,2,3]. Following recent recommendations for uniform terminology, this review uses animal-assisted services (AASs) as the contemporary umbrella term for organised services in which animals are involved to support therapeutic, educational, or other support-related goals [4]. The earlier term AAI is retained only where it appears in historical document titles, organisational terminology, or search strings, because a substantial proportion of the indexed literature remains catalogued under AAI and related terms. In this review, the term therapy dog is retained because it remains widely used in the empirical, organisational, and certification literature. We use it operationally to refer to a specially prepared and assessed dog that participates with a handler or provider in an organised AAS programme. The term does not imply that the dog independently delivers therapy or that a qualified therapist is present in every service, and therapy dogs are distinguished from assistance or service dogs and from emotional support animals. Among the animal species involved in AASs, dogs are the most frequently represented in published research and organised programmes, possibly because of their long co-evolutionary relationship with humans, sociability, responsiveness to human social cues, and trainability [2,5,6,7,8,9]. Their frequent involvement has been associated with their responsiveness to human social cues, trainability, and longstanding roles in human–animal relationships [2,6,9]. Over the past two decades, the evidence base for dog-assisted services has expanded substantially, with studies reporting potential benefits across healthcare, educational, and community settings, including improvements in psychological wellbeing, social engagement, motivation, and stress-related outcomes [2,7,8,10,11,12,13]. As a result, AASs have become increasingly integrated into diverse service contexts in Europe, North America, and other regions.

In contrast to the extensive literature evaluating human outcomes [7,8,10,11,13], considerably less attention has been directed toward how therapy dogs are prepared for participation in AASs [5,6,9]. This gap is notable because preparation practices have direct implications for canine welfare, intervention quality, human safety, and programme sustainability [14,15,16,17]. Therapy dogs are routinely expected to work in environments that may involve unfamiliar people, unpredictable interactions, novel settings, and emotionally charged situations [6,14,17]. Appropriate preparation is therefore essential to ensure that dogs possess not only the behavioural competencies required for intervention work but also the capacity to cope with its physical, social, and emotional demands [6,18,19,20].

The importance of preparation extends beyond operational performance. Contemporary animal welfare science emphasises that welfare encompasses both the prevention of negative experiences and the promotion of positive states, including opportunities for agency, choice, curiosity, social engagement, and other positively valenced experiences [17,19,20,21]. Preparation practices influence whether dogs can participate in AAS in ways that support these welfare outcomes [6,14,15]. From a One Health perspective, canine welfare, human wellbeing, programme effectiveness, and public confidence are closely interconnected [10,15,16]. Inadequate preparation may increase risks related to stress, behavioural difficulties, injury, or disease transmission, whereas effective preparation can contribute to safer, more sustainable, and ethically defensible interventions [15,16,17,22]. Despite its importance, there is currently no widely accepted framework governing the preparation of therapy dogs [6,9,23]. Across the selected European and United States frameworks examined, preparation requirements vary considerably among organisations, professional bodies, and programme providers [6,9,23]. Differences exist in terminology, eligibility criteria, behavioural assessment methods, training expectations, welfare provisions, recertification requirements, and ongoing monitoring practices [1,6,9,23,24]. Consequently, dogs may enter intervention work through markedly different pathways, creating uncertainty regarding minimum standards and limiting comparability across programmes and jurisdictions [5,6,9,23].

The absence of harmonised preparation standards is particularly concerning because therapy dogs are increasingly deployed in complex and sensitive environments, including hospitals, schools, residential care facilities, mental health services, prisons, courts, and crisis response settings [22,25,26,27,28,29]. These contexts differ substantially in sensory load, client behaviour, infection control requirements, emotional intensity, and risks to both humans and dogs [6,15,16,17,22]. While the therapeutic benefits of dog-assisted interventions are now widely documented [7,8,11,13], the systems responsible for preparing and protecting canine participants have not evolved at the same pace [5,6,9,23]. A generic assessment of sociability and obedience may therefore be insufficient to establish readiness for a specific role [5,6]. Instead, preparation should be understood as a lifecycle process in which dogs and handlers are selected, trained, evaluated, supported, monitored, and eventually withdrawn or retired according to evidence-informed criteria [6,14,15,18,30,31]. Consequently, important questions remain regarding what constitutes adequate preparation, how welfare should be monitored throughout service, and whether existing governance models are sufficient to ensure both animal welfare and intervention quality [6,9,14,15,23].

This narrative review addresses these questions by pursuing four objectives and synthesising current evidence and governance practices related to therapy dog preparation in selected European and United States contexts [1,5,6,9,23,24]. First, it maps and compares the current regulatory and certification landscape governing therapy dog preparation, with attention to differences in legal status, assessment procedures, handler requirements, welfare safeguards, and re-evaluation. Second, it synthesises empirical evidence across six core dimensions of preparation: candidate selection [5,6], training methodology [6,14,18,19,20], handler competency [6,9,15,17], welfare monitoring during service [14,15,17,19,20], health and zoonotic risk management [9,15,16], and career lifecycle management [6,14,15,18,30,31]. Third, it identifies priority research gaps that continue to impede the development of evidence-based standard setting, including the lack of longitudinal welfare studies [6,14,15], validated handler competency tools [6,9,17], cross-national governance evaluations [6,9,23], and positive welfare indicators [15,17,19,20,21]. Finally, drawing on the available evidence and existing governance models, it proposes guiding principles for a tiered lifecycle preparation framework that treats therapy dog readiness as a dynamic property of the human–dog team within a specific intervention context rather than as a one-time attribute of the dog alone [5,6,14,15,17,19,20]. The central contribution of this review is therefore integrative. Previous work has examined therapy dog welfare, intervention outcomes, or certification practices as partially separate concerns [5,6,7,8,9,11,13,14,15]. This review brings these strands together by arguing that therapy dog preparation should be conceptualised as a welfare-centred, dyadic, context-specific, and career-long process. In doing so, it aims to support the development of more transparent research reporting, more consistent governance, and more ethically robust standards for the use of dogs in animal-assisted services.

## 2. Materials and Methods

### 2.1. Review Design and Rationale

This article was designed as a structured narrative review of empirical, conceptual, and governance literature concerning the preparation of therapy dogs for AASs. A narrative review design was selected because the literature relevant to therapy dog preparation is methodologically heterogeneous, spanning observational welfare studies, behavioural-assessment research, intervention trials, policy documents, certification standards, legal frameworks, expert consensus papers, and practice guidelines. This heterogeneity makes quantitative synthesis inappropriate, but it is well suited to a critical thematic synthesis aimed at mapping the field, identifying conceptual and governance gaps, and proposing future directions. Accordingly, this review aimed to synthesise evidence across domains that are often treated separately: canine welfare, handler competence, behavioural preparation, certification, governance, and One Health risk management.

### 2.2. Eligibility Criteria

Sources were eligible for inclusion if they addressed one or more of the following topics:

1. selection, screening, temperament assessment, or behavioural evaluation of dogs for AASs, animal-assisted therapy, animal-assisted activities, or animal-assisted education;
2. training, preparation, habituation, or certification of therapy dogs or human–dog teams;
3. welfare assessment of therapy dogs before, during, or after intervention work;
4. handler education, handler competency, human–dog team functioning, or handler recognition of canine stress;
5. governance, regulation, certification, or policy frameworks relevant to therapy dog programmes in Europe or the United States;
6. health, safety, infection control, or zoonotic risk management procedures relevant to dogs participating in AASs;
7. career lifecycle issues, including workload, session frequency, temporary withdrawal from work, retirement, attrition, or burnout-like welfare concerns.

Sources were excluded if they focused exclusively on non-canine species without transferable relevance to therapy dog preparation; addressed assistance dogs, emotional-support animals, or companion dogs without relevance to AAS; reported anecdotal or single-case material without broader conceptual, empirical, or practice relevance; or were non-English publications without an English-language abstract or sufficient English-language metadata for assessment. Because governance and certification practices are often documented outside peer-reviewed literature, grey literature was included when it came from identifiable professional, governmental, international, or certification bodies. Grey literature was not treated as evidence of empirical effectiveness; rather, it was used to describe current standards, policy positions, and governance requirements.

### 2.3. Search Strategy

This was a structured narrative review rather than a systematic review or meta-analysis. No prospective review protocol was registered, and the manuscript does not claim compliance with PRISMA. The review process was structured with reference to the Search, Appraisal, Synthesis, and Analysis domains of the SALSA framework described by Grant and Booth [32]. The completed manuscript was subsequently self-appraised using the Scale for the Assessment of Narrative Review Articles (SANRA) [33]. These frameworks were used to strengthen methodological transparency and reporting quality while preserving the interpretive and integrative purpose of the narrative review design. Searches were conducted in PubMed, Scopus, and Web of Science between February and June 2026, supplemented by Google Scholar, and targeted grey literature retrieval. As shown in Table 1, searches combined terms for the intervention context, the animal participant, and the preparation or governance domain. Search terms were adapted to the syntax of each database. No date restriction was imposed, because the aim was to capture the development of therapy dog preparation practices across the lifespan of the field. Searches were limited to English-language publications or publications with English-language abstracts. The core search string was as follows:

(“therapy dog” OR “therapy dogs” OR “dog-assisted intervention” OR “dog-assisted interventions” OR “canine-assisted intervention” OR “canine-assisted interventions” OR “animal-assisted intervention” OR “animal-assisted interventions” OR “animal-assisted therapy” OR “animal-assisted activities” OR “animal-assisted education”)

AND

(preparation OR training OR selection OR temperament OR assessment OR evaluation OR certification OR accreditation OR governance OR regulation OR welfare OR stress OR cortisol OR oxytocin OR “heart-rate variability” OR handler OR “human-dog team” OR zoonosis OR zoonotic OR hygiene OR retirement OR workload).

Additional targeted searches were conducted for topic-specific evidence using combinations of the following terms: “C-BARQ” AND “therapy dog”; “therapy dog” AND “temperament assessment”; “therapy dog” AND “handler competency”; “animal-assisted intervention” AND “canine welfare”; “therapy dog” AND “cortisol”; “therapy dog” AND “oxytocin”; “animal-assisted intervention” AND “zoonotic risk”; “therapy animal” AND “infection control”; “dog training” AND “aversive methods” AND “welfare”; “therapy dog” AND “retirement”; “therapy dog” AND “burnout”; “animal-assisted intervention” AND “governance”; “therapy dog” AND “certification”; “canine-assisted intervention” AND “guidelines”.

Several contemporary or setting-specific expressions were not included in the original search strategy, including “animal-assisted service”, “dog-assisted service”, “canine-assisted education”, “school dog*”, “wellbeing dog*”, and “well-being dog*”. The search nevertheless incorporated broader and historically established terms, including “animal-assisted intervention”, “animal-assisted therapy”, “animal-assisted activities”, “animal-assisted education”, “therapy dog”, “dog-assisted intervention”, and “canine-assisted intervention”, and was supplemented by Google Scholar and backward and forward citation searching. These overlapping retrieval approaches may have identified some relevant records employing the omitted terminology; however, literature indexed exclusively under these terms may not have been retrieved, particularly literature concerning educational, school-based, or wellbeing-focused services.

### 2.4. Grey Literature and Governance Document Retrieval

Grey literature was included because therapy dog governance is frequently codified in organisational standards, certification manuals, white papers, training documents, and ministerial guidance rather than in peer-reviewed publications. Consistent with the language restrictions specified in the overall search strategy, the grey literature search was conducted in English and was limited to English-language documents or documents containing sufficient English-language information to permit assessment of their relevance and eligibility. Grey literature searches used free-text keywords rather than controlled subject headings. Keyword combinations included (“therapy dog” OR “therapy dogs” OR “animal-assisted service*” OR “animal-assisted intervention*” OR “dog-assisted service*” OR “canine-assisted service*”) AND (standard* OR guideline* OR certification OR accreditation OR governance OR regulation OR legislation OR welfare OR assessment OR “handler training” OR recertification OR workload OR retirement OR hygiene OR zoono*). Targeted searches were conducted for publicly available documents from international organisations, national or regional certification bodies, and governmental authorities. The following categories of documents were eligible:

1. International guidance documents on AAS and animal welfare;
2. Therapy dog certification standards;
3. Handler training or evaluator training requirements;
4. National guidelines, ministerial documents, or regulatory frameworks;
5. Organisational standards of practice;
6. Publicly available welfare, health, hygiene, or zoonotic risk policies;
7. Documents specifying re-evaluation, retirement, workload, or incident reporting requirements.

Documents were included only when they were publicly accessible, attributable to an identifiable organisation or authority, and directly relevant to therapy dog preparation or governance. Where documents referred primarily to assistance dogs rather than therapy dogs, they were included only if they had clear implications for AAS governance, public access policy, canine welfare, or comparative regulatory development. Assistance dog frameworks were not treated as equivalent to therapy dog certification systems.

The governance analysis focused on selected publicly available frameworks from the United States and selected European jurisdictions. These frameworks were included as illustrative examples of different governance arrangements, including voluntary certification, international professional guidance, nationally coordinated or legally embedded systems, charity-led models, and project-based guidance, rather than as a representative sample of either region. The search was targeted and did not involve a systematic country-by-country mapping of all European Union Member States or all countries geographically located in Europe. Accordingly, the absence of a jurisdiction or framework from the synthesis should not be interpreted as evidence that no relevant governance procedures exist. Consistent with the language restriction described in Section 2.3, the grey literature search was conducted in English; therefore, relevant governance documents published exclusively in national languages may not have been identified. Grey literature sources included the IAHAIO White Paper [1], the Pet Partners Standards of Practice in Animal-Assisted Interventions, the EU Erasmus+ Human Dog Team Project handbook, ministerial documentation from Austria and Italy, and AAS guidance from the American Veterinary Medical Association. No date restriction was imposed.

### 2.5. Data Charting and Extraction

Information from included sources was charted using a structured extraction framework developed from the review objectives and refined inductively during reading. For peer-reviewed empirical and review sources, the following information was extracted where available:

1. Author, year, country, and publication type;
2. Study design or review type;
3. AAS context or intervention setting;
4. Dog population and demographic information;
5. Selection, training, or certification procedures;
6. Handler characteristics or handler-training requirements;
7. Welfare measures, including behavioural, physiological, and positive-welfare indicators;
8. Health, hygiene, or zoonotic risk procedures;
9. Workload, rest, retirement, or lifecycle-management information;
10. Key findings relevant to preparation, welfare, governance, or research gaps;
11. Limitations relevant to interpretation.

For governance and certification documents, the following information was extracted:

1. Organisation or jurisdiction;
2. Legal or voluntary status;
3. Minimum dog eligibility criteria;
4. Health screening requirements;
5. Behavioural or temperament assessment procedures;
6. Handler training requirements;
7. Training method expectations, including any position on aversive methods;
8. Welfare monitoring requirements;
9. Hygiene and zoonotic risk management requirements;
10. Incident-reporting procedures;
11. Re-evaluation or recertification requirements;
12. Retirement, withdrawal, or workload guidance.

This extraction framework was used to construct the governance comparison and to identify recurring preparation domains across the literature.

### 2.6. Thematic Synthesis

Given the heterogeneity of study designs, populations, measurement approaches, and governance contexts in the included literature, meta-analysis was not appropriate. Findings were synthesised narratively using a thematic approach. Initial themes were based on the review objectives and on the six preparation dimensions: candidate selection, training methodology, handler competency, welfare monitoring, health, safety, zoonotic risk management, and career lifecycle management.

The final thematic structure was as follows:

1. Governance and certification landscape;
2. Candidate selection and temperament assessment;
3. Training methodology and welfare implications;
4. Handler competency and the human–dog dyad;
5. Welfare monitoring during active service;
6. Health, safety, and zoonotic risk management;
7. Career lifecycle management;
8. Priority research gaps and future directions.

These themes were analysed in relation to three cross-cutting frameworks: the Five Domains Model of animal welfare, One Health, and dyadic human–dog team functioning. This approach allowed the review to move beyond a descriptive summary of certification practices and toward a welfare-centred interpretation of preparation as a career-long process.

### 2.7. Governance Comparison

A comparative governance matrix was developed to examine publicly available standards and regulatory frameworks across selected organisations and jurisdictions in Europe and the United States. The comparison focused on whether each framework specified requirements or guidance in five areas:

1. Dog evaluation or aptitude assessment;
2. Handler training or handler competency assessment;
3. Welfare monitoring or welfare safeguards;
4. Legal, regulatory, or voluntary status;
5. Re-evaluation, recertification, or ongoing assessment.

The frameworks were selected because they were publicly accessible, sufficiently detailed to permit structured extraction, and illustrative of different governance arrangements, including voluntary certification systems, international guidance frameworks, nationally coordinated or legally embedded approaches, charity-led models, and European project-based guidance. The matrix was not intended to provide an exhaustive or representative mapping of all governance arrangements in the United States or Europe, and the absence of a country, jurisdiction, or framework should not be interpreted as evidence that no relevant procedures exist. The purpose of the comparison was not to rank organisations or jurisdictions but to identify structural variation in preparation requirements and to highlight areas in which harmonisation or further empirical evaluation may be needed.

### 2.8. Assessment of Evidence Strength and Limitations

Formal risk-of-bias assessment was not conducted because this was a narrative review that included heterogeneous empirical studies, reviews, policy documents, and organisational standards. Instead, evidence was interpreted according to source type, methodological transparency, directness of relevance, sample size, ecological validity, and consistency with other findings. Empirical studies involving therapy dogs in AAS contexts were treated as most directly relevant to welfare and preparation questions. Studies of companion dog training, assistance dog selection, or general canine behaviour were included only when their findings were transferable to therapy dog preparation. Governance documents were analysed as evidence of standards and policy requirements, not as evidence that those standards produce improved welfare or intervention outcomes. The principal limitations of the method are the non-systematic nature of narrative synthesis, the likelihood that some local or non-English governance documents were not captured, the uneven public availability of organisational standards, and the limited capacity to compare certification systems where internal training or monitoring procedures are not publicly described.

### 2.9. Ethics and Data Availability

This review did not involve new data collection from human participants or animals. Ethical approval was therefore not required. No new datasets were generated. All sources used in the review were publicly available published articles, organisational documents, or policy materials.

## 3. Results

### 3.1. Overview of the Evidence Synthesis

Across the sources reviewed, six recurrent preparation domains emerged: candidate selection, training methodology, handler competency, active service welfare monitoring, health and zoonotic risk management, and career lifecycle management. These domains are interdependent, such that weaknesses in one area can undermine welfare or safety in another.

Three cross-cutting findings were identified. First, the selected governance frameworks identified in the United States and selected European jurisdictions varied substantially in their legal status, scope, handler requirements, welfare provisions, and re-evaluation procedures. Second, empirical evidence is strongest for welfare monitoring during active service and weakest for career lifecycle management, handler competency validation, and comparative governance evaluation. Third, current preparation standards often emphasise observable behavioural outcomes while giving less systematic attention to training history, positive welfare, canine agency, context-specific readiness, and longitudinal welfare trajectories. The following subsections present the results of the thematic synthesis.

### 3.2. Governance and Certification Landscape

Across the selected frameworks, the governance of therapy dog preparation varied substantially in legal status, scope, and enforceability. Unlike assistance dogs, which are increasingly supported by statutory frameworks in many jurisdictions, the therapy-dog frameworks examined in this review were primarily based on voluntary certification systems, organisational standards, professional guidance, or selected nationally coordinated and legally embedded arrangements [6]. Variation was identified in candidate selection, handler education, welfare safeguards, health screening, and re-evaluation. Table 2 presents an illustrative comparison of selected publicly available frameworks from the United States and selected European jurisdictions; it is not intended as a comprehensive or representative overview of governance in either region.

International frameworks provide important ethical and professional guidance but not a single enforceable global standard. IAHAIO’s 2018 White Paper, ISAAT accreditation activity, and the Pet Partners Standards of Practice all emphasise animal welfare, handler responsibility, assessment, and risk management, but their implementation depends on adoption by local organisations or institutions [1,24,34]. This helps explain the heterogeneity observed among the programmes and jurisdictions included in the review.

Among the selected United States frameworks, therapy-dog preparation was governed primarily through voluntary organisations and institutional policies rather than statute. Publicly available materials from these organisations indicate considerable variation in eligibility requirements, team evaluation, handler education, re-evaluation intervals, and welfare monitoring expectations. Pet Partners represents one of the more structured voluntary models, requiring handler training and assessment and an in-person roleplay-based team evaluation every two years, while also framing handlers as responsible advocates for their animals [24]. Therapy Dogs International (TDI) similarly evaluates dog–handler teams, but its public materials indicate that applicants are not required to take therapy dog classes and that TDI does not offer or sanction such classes [35]. The American Kennel Club (AKC) therapy dog title programme should also be distinguished from therapy dog certification because it recognises visits completed through AKC-recognised organisations rather than functioning as a standalone readiness certification system [36]. Taken together, these selected United States examples illustrate flexibility and broad participation but also unevenness in preparation, handler training, welfare monitoring, and health risk management, consistent with the variability documented by Serpell et al. [9].

The selected European frameworks likewise illustrate heterogeneity, including more formalised or nationally coordinated arrangements alongside project-based and charity-led approaches. Italy illustrates national coordination through its reference centre and guideline framework for AAS, which defines intervention categories, professional roles, and training requirements within a multidisciplinary and One Health-oriented model [37]. Austria provides another comparatively structured model, with legal provisions for assistance and therapy companion dogs and assessment regulations that require training of both dog and handler, a positive expert assessment, and explicit reference to animal welfare and One Health principles [38]. Other European examples illustrate less formal but still influential governance pathways, including project-based professionalisation through the Human Dog Team handbook [39,40] and charity-led assessment models such as Pets As Therapy in the United Kingdom [41]. Germany’s Teilhabestärkungsgesetz, although not specific to therapy dogs, has broader implications for canine-assisted services and has stimulated discussion regarding governance in healthcare settings, while developments in Belgium and Sweden indicate growing recognition of the need for more consistent standards [23]. At the European level, this heterogeneity is further reflected in variation across national guidelines and legal frameworks for canine-assisted interventions [23]. These examples should not be interpreted as representative of Europe as a whole. Relevant governance procedures in other jurisdictions may not have been captured, particularly where documentation was available only in national languages or was not publicly accessible.

Taken together, the governance comparison indicates that no single reviewed model fully addresses all dimensions of therapy dog preparation. Voluntary systems may offer accessible and scalable team evaluation but vary in training requirements and ongoing welfare monitoring; nationally coordinated systems can provide greater consistency and accountability but require institutional infrastructure and implementation mechanisms; and international frameworks offer valuable guidance but depend on local uptake. The main comparative finding is therefore not that one jurisdiction has solved the problem but that the reviewed frameworks differ structurally across legal, professional, and organisational contexts.

### 3.3. Candidate Selection: Temperament, Health, and Contextual Suitability

Candidate selection in current AAS practice generally combines behavioural screening, health review, and an assessment of whether the dog is suited to the intended working context rather than to AAS work in general [9,42]. Survey data from U.S. therapy dog organisations indicate that most programmes require formal written eligibility criteria and an in-person behavioural evaluation before registration, with many also requiring minimum age thresholds for participation [9]. IAHAIO guidance similarly states that dogs should be individually assessed by an experienced evaluator for the temperament and behaviour required in specific settings, noting that suitability may differ across contexts such as prisons, hospitals, or visits with frail older adults [1]. This reflects a broader shift from generic certification toward context-dependent suitability assessment.

Current assessment practice also treats temperament as a multidimensional construct that includes sociability, emotional stability, touch tolerance, environmental resilience, and recovery after stimulation rather than as a single “friendly” disposition [6]. In practice, many organisations use formal behavioural or aptitude tests designed to simulate interactions likely to occur during visits, although the content and depth of these tests vary across providers [9]. Peer-reviewed work further suggests that stronger selection protocols are likely to be multi-stage, integrating owner report information, direct behavioural observation, and practical team evaluation [5,43]. Owner report instruments such as the Canine Behavioral Assessment and Research Questionnaire (C-BARQ) have shown potential value in identifying fearfulness, excitability, and aggression-related traits relevant to therapy dog suitability, especially when used as a preliminary screening tool rather than a standalone decision instrument [43,44].

Health screening is also a routine component of current selection practice. American Veterinary Medical Association guidance recommends preventive veterinary care, vaccination, parasite control, selected screening for infectious and parasitic diseases, and behavioural assessment as part of wellness oversight for animals involved in AASs [42]. IAHAIO likewise recommends veterinary health assessment before admission to an AAI programme, annual veterinary wellness examinations, prophylactic parasite control, and exclusion of animals that are ill, recovering from surgery, pregnant, or showing unexplained behavioural change until cleared for participation [1]. These recommendations indicate that current selection practice is not limited to identifying dogs that are socially tolerant; it also seeks to exclude dogs whose health status, pain, transport intolerance, or poor recovery could compromise welfare or increase risk during intervention work [1,42].

A further feature of current practice is increasing emphasis on team-based and setting-relevant assessment. IAHAIO recommends that evaluation be appropriate to the specific setting, that handlers normally be the dog’s owner or a familiar approved handler, and that dogs be familiarised with the facility before visits begin [1]. Practical guidance for educational and healthcare programmes similarly emphasises preparatory visits, environmental familiarisation, and observation of the team in realistic working conditions [22,26]. Taken together, current candidate selection is best characterised as a staged process in which temperament, health, handler familiarity, and contextual fit are assessed together rather than as isolated criteria [1,6,9].

### 3.4. Training Methodology and Context-Specific Preparation

Current training practice in AASs is increasingly centred on reward-based, relationship-focused, and humane methods [1,9,24]. IAHAIO explicitly states that only relationship- and reward-based training methods should be used in AAS preparation and that handlers should complete training and behavioural modification programmes that are free from negative stress and support advocacy for the dog during visits [1]. Survey data from U.S. therapy dog organisations are broadly consistent with this direction: most organisations reported written policies on acceptable training methods, and many specifically prohibited coercive equipment such as prong collars, choke collars, or electronic collars, while requiring positive reinforcement approaches [9]. Current practice therefore appears to be moving away from aversive control and toward methods intended to support both reliable behaviour and canine welfare [6].

At the same time, the peer-reviewed canine training literature remains relevant because AAS-specific research on training methodology is still limited [6,14]. Reviews of companion dog training consistently report welfare concerns associated with aversive methods, including increased fear, stress-related behaviour, and poorer human–dog interaction quality, while reward-based approaches are more consistent with welfare-protective training principles [45,46,47]. In the AAS context, this matters because behavioural compliance alone may not indicate genuine readiness for close-contact therapeutic work. A dog may appear controlled while experiencing distress, avoidance, or reduced willingness to engage [6,18]. For this reason, current welfare-oriented training practice increasingly emphasises not only what the dog can do but how the dog was trained and whether the dog can work voluntarily without coercion [14,17,19,20].

Context-specific preparation is another clear feature of current practice. IAHAIO recommends that evaluation and preparation be appropriate to the actual setting, that handlers visit facilities with the dog before work begins, and that initial visits involve accompaniment by an established team or evaluator while the handler remains alert to stress signals [1]. This aligns with wider guidance in educational and healthcare settings that dogs should be prepared for the specific sensory, social, and procedural demands of the environment in which they will work, rather than relying solely on generic obedience or temperament assessment [6,22,26,28]. In practice, this may include gradual exposure to wheelchairs, mobility aids, medical equipment, crowded corridors, children’s movement patterns, loud noises, unusual flooring, prolonged stationary behaviour, or tactile interaction from unfamiliar people, depending on the intended placement [1,6,22,26].

Current practice also increasingly recognises that preparation should include the dog’s ability to disengage. IAHAIO states that animals should willingly engage in interactions and must not be forced, with retreat opportunities and session termination required if stress cannot be reduced effectively [1]. This principle is important because welfare-protective preparation is not achieved simply by teaching the dog to remain still or compliant. Instead, current best practice increasingly frames training as preparation for safe, voluntary, and sustainable participation, supported by handler recognition of stress, provision of breaks, and responsiveness to signs of fatigue or reluctance [1,9,14,15,17].

Taken together, current training methodology in AAS is best described as a staged, humane preparation process that combines reward-based learning, familiarisation with realistic working conditions, and handler education in welfare-sensitive decision making [1,6,9,24]. It is not limited to teaching commands. Rather, it aims to prepare a dog–handler team to function reliably in a specific intervention context while protecting canine welfare and preserving the dog’s opportunity to withdraw when needed [6,14,15,20].

### 3.5. Handler Competency and the Human–Dog Dyad

The evidence synthesis strongly supports a dyadic model of therapy dog preparation, in which therapy work is conceptualised not as the performance of a dog in isolation but as the joint functioning of a human–dog team [6,9,24,48]. The handler is arguably the most influential determinant of therapy dog welfare and effectiveness because handler behaviour shapes the dog’s exposure to stimuli, emotional regulation, safety, opportunities for rest, and capacity to disengage from stressful interactions [14,15,17]. Handler decisions shape candidate selection, training practices, workload management, interaction quality, and the recognition and management of stress-related behaviours [6,49,50,51]. Consequently, therapy dog preparation must be understood as the preparation of the human–dog dyad rather than the dog alone [6,9,48].

Within this framework, handler competency emerges as a core domain rather than a secondary or administrative requirement. Handler behaviour directly influences the dog’s exposure to stimuli, emotional regulation, safety, opportunities for rest, and capacity to disengage from stressful interactions. Despite this central role, handler competency remains insufficiently studied and poorly operationalised in existing certification systems. Emerging evidence further reinforces the importance of adopting a dyadic perspective. Compatibility between handler and dog personalities has been shown to influence interaction quality beyond the contribution of either individual alone [48]. This finding suggests that therapy dog certification processes should assess dyadic functioning rather than evaluating dogs and handlers independently. At the same time, research indicates a potential disconnect between handler awareness and practice. Handlers frequently identify themselves as a primary source of welfare risk while simultaneously demonstrating a positive bias in their assessment of their own dogs’ welfare [50]. This highlights the need for more objective and structured approaches to evaluating handler competency.

Four core competencies emerge as particularly critical for effective and ethical therapy dog practice. First, handlers require advanced canine stress recognition skills. They must be able to identify both overt and subtle indicators of discomfort, including gaze aversion, lip licking, yawning, panting unrelated to heat, body stiffening, displacement behaviours, avoidance, tucked tail, lowered posture, heightened vigilance, changes in responsiveness, and reductions in affiliative behaviour. However, recognition of these signals remains inconsistent. Subtle stress indicators are frequently overlooked or misinterpreted, particularly by experienced handlers who may become habituated to their dogs’ behavioural patterns over time [51]. Similarly, studies of Italian handlers found that while basic stress recognition skills were present, there were substantial gaps in the implementation of stress management strategies and environmental modification techniques [49].

Second, handlers require decision making authority and confidence. The ability to identify stress is insufficient if handlers do not feel empowered to act [17,50]. Effective practice requires that handlers can pause, redirect, modify, or terminate sessions when necessary [1,14,17,24]. Training programmes should explicitly prioritise canine welfare over client expectations, institutional pressures, or goals related to visit completion [6,14,15,52].

Third, handlers must possess environmental management skills. These include the ability to position the dog to avoid crowding, regulate approach speed, manage leash tension, create and enforce rest opportunities, monitor environmental conditions such as temperature and floor surfaces, prevent inappropriate or intrusive touch, and structure predictable entry and exit routines [6,17,22,24]. These competencies are especially critical in high-risk or high-demand environments, such as those involving children, individuals with cognitive impairments, psychiatric distress, mobility aids, or elevated sensory stimulation [6,22,26,27,28,29].

Fourth, handlers require reflective capacity and awareness of cognitive bias. As demonstrated by Mignot et al. [50], handlers may simultaneously recognise general welfare risks and underestimate those affecting their own dogs. This suggests that reliance on self-report alone is insufficient. Instead, handler evaluation should incorporate structured welfare checklists, external observation, and periodic reassessment to ensure more objective and reliable judgements.

Despite the recognised importance of these competencies, there remains considerable heterogeneity in how human–dog interaction quality is conceptualised and measured. Moreover, positive behavioural indicators, such as play behaviour, affiliative gestures, and dog-initiated contact, are consistently under-measured relative to stress-related indicators. This imbalance limits the field’s ability to capture the full spectrum of welfare and interaction quality.

Overall, the literature converges on the conclusion that handler training is a critical yet underdeveloped component of therapy dog preparation. Existing certification systems, which often rely on single-session behavioural assessments or simulated visits, do not consistently evaluate handler knowledge, stress interpretation, ethical decision making, or environmental management under ecologically valid conditions. Advancing the field will require the development of validated assessment tools, standardised competency frameworks, and empirically informed training protocols that explicitly target the functioning of the human–dog dyad.

### 3.6. Welfare Monitoring During Active Service

Welfare monitoring during active service represents the most empirically developed dimension of therapy dog preparation. Yet findings remain mixed and characterised by small sample sizes, methodological heterogeneity, and a limited number of longitudinal studies. Overall, evidence indicates that therapy dog welfare is highly variable and context-dependent, underscoring the need for theoretically grounded and multimodal assessment approaches.

A useful conceptual framework is provided by the Welfare Quality approach, which evaluates welfare across four domains: nutrition, environment, health, and behaviour [15]. When applied to AAS, this framework suggests that therapy dogs may encounter challenges across all domains, including suboptimal environmental conditions, workload demands, and behavioural stressors. These principles align closely with the Five Domains Model of animal welfare, which emphasises not only the prevention of negative states but also the promotion of positive affective experiences [19]. Together, these frameworks reinforce the need to move beyond narrow definitions of welfare centred on stress reduction toward a more comprehensive understanding of animal wellbeing.

Early research on therapy dog welfare focused predominantly on salivary cortisol as a biomarker of hypothalamic–pituitary–adrenal (HPA) axis activation. Findings from these studies have been mixed. Some studies reported cortisol increases associated with therapy sessions, transportation, or environmental demands [25,53], identifying potential welfare risks that remain underrepresented in existing certification frameworks. In contrast, other studies found no significant cortisol elevations among experienced therapy dogs working in highly structured settings, such as paediatric oncology environments [54], while reductions in cortisol have been observed in certain intervention contexts, such as shelter dogs participating in structured programmes [25].

These apparently divergent findings should not be interpreted as contradictory but rather as evidence that welfare outcomes are shaped by multiple interacting variables. These include individual dog characteristics, prior experience, handler behaviour, session structure, environmental novelty, transport conditions, availability of rest, and the degree of control the dog has over interactions. Cortisol alone is therefore insufficient as a standalone welfare indicator. It is influenced not only by stress but also by arousal, physical activity, circadian rhythms, novelty, and individual physiological variation. Similarly, behavioural indicators are inherently limited, as stress signals may be subtle, context-dependent, or actively suppressed.

Recent advances have expanded welfare assessment beyond cortisol-based approaches. Glenk and Foltin’s review [14] identified a range of additional physiological indicators, including salivary oxytocin, heart rate variability, respiratory rate, and tympanic membrane temperature, as potentially informative measures of welfare. However, the interpretation of these indicators remains complex, and their utility depends on careful integration with behavioural and contextual data. In particular, emerging interest in salivary oxytocin and vasopressin reflects a shift toward assessing positive welfare states and affiliative processes. These biomarkers are associated with social bonding and positive affect, but methodological challenges—including assay reliability, contextual sensitivity, and individual variability—currently limit their routine application [48].

This shift is further reflected by the growing adoption of multimodal welfare frameworks. Such approaches are especially important because positive emotional states are difficult to infer from any single indicator and require convergent evidence from behaviour, physiology, context, and repeated observation [21]. De Winkel et al. [55] propose an integrative approach that combines physiological measures with behavioural indicators of both negative and positive welfare. In this framework, assessment extends beyond the absence of distress to include evidence of flourishing, such as play behaviour, affiliative interactions, voluntary engagement, and dog-initiated contact. Notably, earlier research identified a systematic imbalance in the literature, with positive welfare indicators being under-measured relative to stress-related outcomes.

Synthesising current evidence, welfare monitoring during active therapy work should be multimodal and include several key components. These comprise the following:

1. Behavioural indicators of stress (e.g., avoidance, displacement behaviours, vigilance, startle responses, panting, body tension, reduced responsiveness);
2. Behavioural indicators of positive welfare (e.g., relaxed posture, voluntary approach, play, affiliative contact, exploratory behaviour);
3. Physiological indicators where feasible (e.g., cortisol, heart rate variability, respiratory rate, oxytocin/vasopressin);
4. Recovery indicators (e.g., post-session rest, sleep quality, appetite, behaviour at home, willingness to engage in future work); and
5. Contextual variables (e.g., session duration, number of clients, type of interactions, noise levels, transport demands, environmental conditions, and handler behaviour).

Importantly, welfare assessment should occur across multiple time points rather than being confined to single-session observations. Monitoring should include pre-session anticipation, in-session behaviour, immediate post-session responses, and longer-term recovery at home. This temporal dimension is critical, as cumulative welfare strain may not be evident during a session itself. For example, a dog that appears compliant during interactions may exhibit avoidance behaviours prior to sessions, fatigue afterward, disrupted sleep, or reluctance to re-engage in therapy work. Such patterns are rarely captured within one-time certification or evaluation procedures. In summary, the literature consistently indicates that welfare monitoring in therapy dog programmes must extend beyond single indicators and isolated time points. Although the empirical base is expanding, methodological inconsistencies and conceptual limitations remain. Future progress will depend on the development of standardised, multimodal, and longitudinal assessment frameworks that integrate physiological, behavioural, and contextual data, aligned with contemporary models of animal welfare and sensitive to the dynamic and dyadic nature of therapy work.

### 3.7. Health, Safety, and Zoonotic Risk Management

Current practice in AAS treats health and zoonotic risk management as part of routine programme delivery, particularly in settings involving vulnerable populations. A One Health perspective is increasingly used because canine health, human health, and environmental hygiene are interdependent in therapy dog work [15,16].

Most programmes require routine veterinary oversight, including vaccination review, parasite control, and exclusion of dogs with signs of illness, pain, dermatological disease, or gastrointestinal or respiratory symptoms [9,16,17,56]. This reflects evidence that clinically normal dogs may still carry zoonotic organisms, so preventive screening and illness exclusion policies are preferred to visual inspection alone [57,58].

Current practice also relies on written hygiene protocols. Common measures include appropriate grooming, clean equipment, mandatory hand hygiene before and after contact, and setting-specific restrictions that prevent dogs from contacting wounds, invasive devices, medical equipment, or food areas [56,57,59]. In higher-risk settings, raw-fed dogs are often discouraged or excluded because raw diets may increase shedding of zoonotic pathogens [60,61,62].

Overall, current practice centres on veterinary clearance, hygiene, contact restrictions, illness exclusion, diet-related precautions, and incident reporting, but these elements are not standardised consistently across organisations and jurisdictions [9,15,16]. This inconsistency supports treating health and zoonotic risk management as a core component of therapy dog preparation rather than an optional add-on.

### 3.8. Career Lifecycle Management

Career lifecycle management remains one of the least empirically developed domains of therapy dog preparation [14,63]. Although most certification systems assess readiness at entry and at periodic intervals, few provide evidence-based guidance on workload limits, cumulative stress, temporary withdrawal, reduced duties, or retirement [30,31].

The available literature suggests that therapy dog work can create cumulative demands through travel, environmental novelty, social interaction, unpredictable handling, and emotional arousal [5,27]. These demands are not uniform: a dog may tolerate low-intensity sessions for years yet experience strain in high-contact healthcare environments, or may remain comfortable during brief hospital visits but become fatigued after lengthy travel or multiple sessions in a single day [22,26,28]. Career management therefore needs to be individualised and based on repeated welfare assessment rather than fixed activity categories.

Relevant lifecycle variables include age at entry into service, physical maturity and health status, previous socialisation and training history, session duration and frequency, number and type of clients per session, transport duration and conditions, environmental intensity, recovery time between visits, changes in health, pain, sensory function, or mobility, behavioural signs of reduced motivation or increased avoidance, and the handler’s ability to modify or retire the dog from work. Retirement should be framed as a welfare-positive transition rather than a failure. Dogs should be withdrawn temporarily or permanently when they show persistent reluctance, reduced recovery, escalating stress signals, declining health, pain, sensory impairment, reduced resilience, or loss of enjoyment. Cortesi et al. [49] provide emerging evidence that handlers recognise stress and burnout-like concerns in dogs involved in AAS, but this area remains underdeveloped. There is still insufficient empirical evidence to define universal workload thresholds or retirement ages, so programmes often rely on handler judgement, organisational custom, or informal norms [49]. An overview of the evidence synthesis across core therapy dog preparation domains is presented in Table 3.

### 3.9. Principal Research Gaps Identified

Seven research gaps were identified across the evidence base [6,14,15]. First, there is no robust comparative evidence showing whether formal national regulatory systems, voluntary certification systems, international standards, or hybrid models produce better welfare, safety, or intervention outcomes [6,9,23]. Second, therapy dog selection tools require further validation. Candidate screening should be tested prospectively against long-term welfare, retention, incident rates, and handler-reported suitability rather than only against short-term pass–fail outcomes [6,43,64,65]. Third, training method research specific to therapy dogs remains limited. Evidence from companion dog training strongly supports welfare concerns about aversive methods, but therapy dog-specific studies are needed to evaluate how training history affects performance, stress signalling, welfare, and safety in AAS environments [14,45,46,47]. Fourth, handler competency remains poorly operationalised. There is no widely validated tool for assessing handler stress-recognition accuracy, welfare decision-making, environmental management, or the ability to support canine agency, voluntary engagement, and positively valenced participation [20,21,49,50,51]. Fifth, welfare monitoring remains methodologically inconsistent. Cortisol, oxytocin, heart rate variability, behavioural indicators, and handler reports are all potentially useful, but no agreed multimodal minimum dataset exists for therapy dog welfare research or certification [15,17,48,53,54]. Sixth, career lifecycle management lacks empirical foundations. Longitudinal studies are needed to identify early indicators of cumulative fatigue, burnout-like states, declining motivation, and appropriate timing of retirement [14,49,63]. Seventh, reporting standards require broader adoption. Recent dog-assisted intervention reporting extensions to SPIRIT and CONSORT address the problem of inconsistent reporting in dog-assisted intervention trials and are directly relevant to future AAS research transparency [66]. Future empirical studies should routinely report dog demographics, selection procedures, training methods, handler qualifications, intervention setting, session structure, welfare indicators, health protocols, and withdrawal criteria [5,66].

## 4. Discussion

This review identifies a clear mismatch in the field: therapy dogs are increasingly involved in healthcare, education, social care, and community settings, yet the systems that prepare, monitor, and protect them remain unevenly developed [6,7,8,9,13,23]. Governance is fragmented across jurisdictions and organisations. Among the selected frameworks identified in this review, the United States examples were predominantly voluntary certification or registration models, whereas the European examples included nationally coordinated, legally embedded, charity-led, and project-based arrangements [23,37]. These observations should not be interpreted as a comprehensive characterisation of either region. In particular, the European findings reflect selected countries and organisations captured through a targeted grey literature search and may omit procedures published only in national languages or not readily accessible online. International frameworks such as IAHAIO and ISAAT provide important guidance, but they are not uniformly enforceable [1,34]. As a result, preparation continues to vary in candidate selection, handler education, welfare monitoring, health screening, and re-evaluation.

This review also shows that therapy dog preparation should not be treated as a single pre-service test. A more accurate model is a lifecycle process that includes candidate selection, health screening, reward-based and context-specific training, handler competency, active-service welfare monitoring, health and zoonotic risk management, workload regulation, and retirement planning [5,6,15,16,49]. These elements are interdependent: a suitable dog may still be placed at risk by poor handler judgement, while a competent handler may be constrained by a certification system that does not require structured welfare monitoring or setting-specific preparation. Readiness is therefore dynamic, changing across the dog’s working life as health, motivation, behaviour, and work demands change [15,49].

Readiness also belongs to the human–dog team in context, not to the dog alone. Dog temperament and health remain essential, but handler skill is equally central because the handler must recognise stress, manage interactions, create opportunities for rest, advocate for the dog, and withdraw from unsuitable situations when needed [17,48,50,51]. This dyadic view matters because assessment should capture whether the team can function safely, ethically, and flexibly in real intervention settings, not only whether the dog can tolerate contact or obey cues. It also aligns with the broader welfare argument that positive welfare, choice, curiosity, social engagement, and agency matter alongside the absence of overt distress [19,20,21].

The strongest current evidence concerns welfare monitoring during active service, while the weakest evidence concerns lifecycle management, comparative governance evaluation, validated handler competency assessment, and positive welfare indicators. Studies have improved understanding of cortisol, behaviour, handler perception, and contextual stressors, but the field still lacks longitudinal evidence following therapy dogs from selection through retirement [14,25,49,52,54,66]. This leaves workload decisions, temporary withdrawal, and retirement planning too dependent on informal judgement rather than evidence-based standards. The practical implication is that welfare assurance must extend beyond certification and into the dog’s full working life.

The Five Domains Model offers a useful welfare framework because it integrates nutrition, physical environment, health, behavioural interactions, and mental state rather than treating welfare as the absence of distress alone [19]. In therapy dog work, this means attention to diet, hydration, parasite control, fitness, transport, noise, flooring, rest spaces, handling, choice, play, affiliative contact, and opportunities to disengage [15,19]. It also means recognising agency as a welfare issue: dogs cannot consent in the human sense, but they can signal willingness, avoidance, preference, and disengagement, and those signals should be treated as meaningful communication rather than disobedience [17,20]. For the same reason, welfare assessment should go beyond stress-only indicators such as cortisol, heart rate, panting, or avoidance and include positive welfare indicators such as voluntary approach, relaxed contact, play, curiosity, exploration, affiliative interaction, and rapid recovery [14,19,20,21,48,52,54].

The governance findings suggest that harmonisation should focus on minimum welfare and competency standards rather than a single international certification model. Because legal systems, professional cultures, healthcare structures, and animal welfare policies differ across countries, identical regulation is unlikely; however, core requirements are still feasible, including documented candidate selection, veterinary screening, temperament and environmental-suitability assessment, discouragement of aversive methods, handler education, team assessment, setting-specific preparation, welfare monitoring, visit exclusion rules, incident reporting, periodic re-evaluation, and workload and retirement guidance [1,5,6,14,16,17,23,24,27,34,38,43,44,45,46,47,48,49,50,51,54,56].

### 4.1. Proposed Tiered Lifecycle Framework for Therapy Dog Preparation

**Figure 1:**
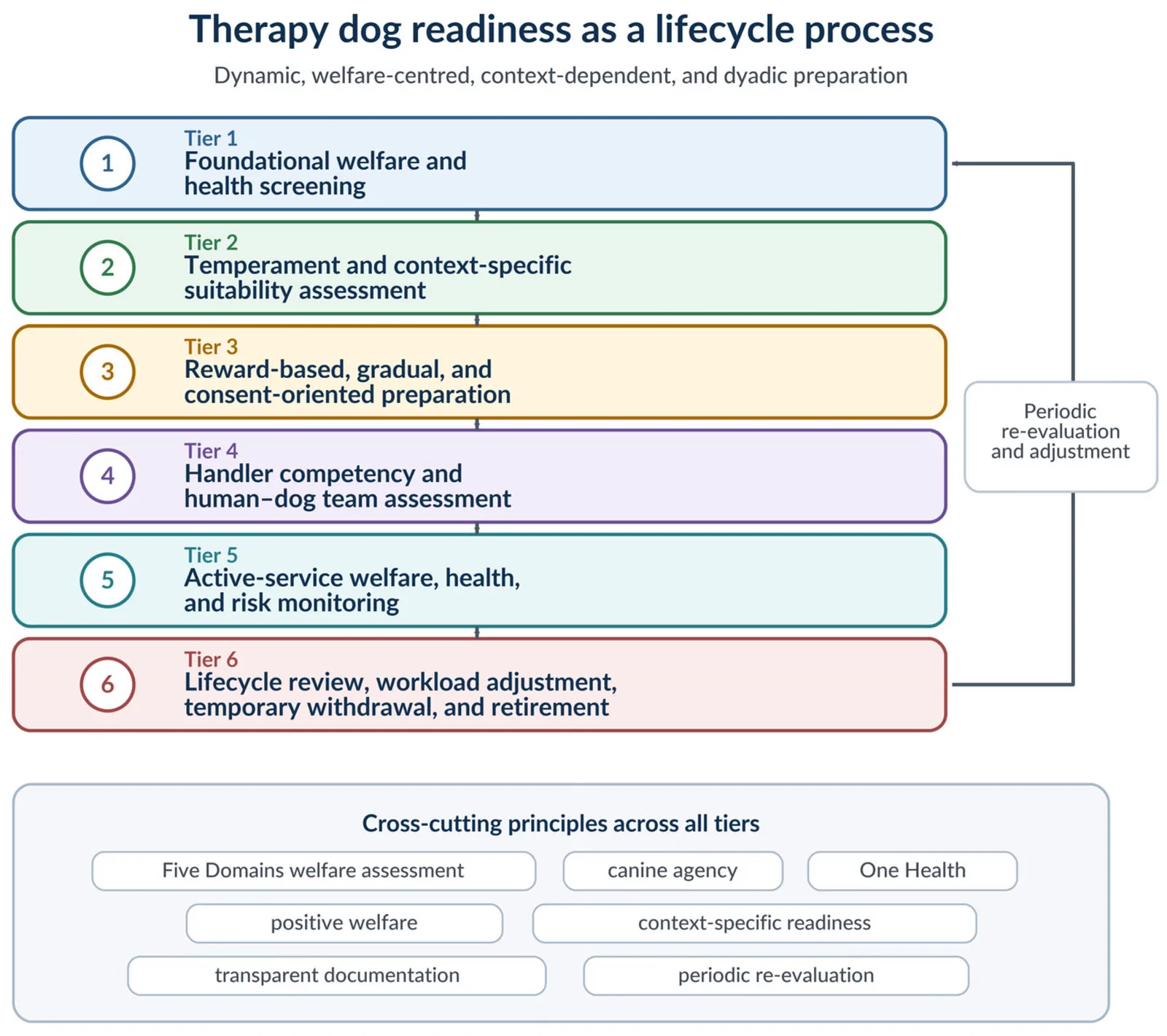
Proposed tiered lifecycle framework for therapy dog preparation and welfare assurance. The six tiers describe a staged process from foundational screening through active-service monitoring and lifecycle review. Cross-cutting principles apply across all tiers: Five Domains welfare assessment, canine agency, One Health, positive welfare, context-specific readiness, transparent documentation, and periodic re-evaluation. The cross-cutting principles reflect contemporary welfare and sentience approaches that integrate negative-state prevention with positive welfare, agency, voluntary engagement, and multimodal assessment. Source: Authors’ own work.

Taken together, the evidence supports a tiered lifecycle view of therapy dog readiness. We therefore propose a six-tier lifecycle framework for therapy dog preparation that treats readiness as a dynamic, welfare-centred, and context-dependent property of the human–dog team rather than a one-time certification outcome. This framework is grounded in a positive-welfare understanding of sentience, in which readiness requires not only reduced risk of distress but also evidence of voluntary, engaged, and positively valenced participation [21]. A conceptual overview of the proposed tiered lifecycle framework for therapy dog preparation and welfare assurance is provided in Figure 1.

In the first tier, dogs should undergo foundational eligibility screening that considers physical health, vaccination status, parasite control, absence of unmanaged pain or illness, and behavioural stability. The second tier should assess temperament and context-specific suitability, recognising that a dog may be appropriate for one type of setting but not for another, depending on factors such as noise, crowding, interaction intensity, and client vulnerability. The third tier should focus on reward-based, consent-oriented preparation, while the fourth tier should evaluate handler competency and the quality of the human–dog team, including the handler’s ability to recognise stress signals and end a session when needed. The fifth tier should involve welfare and risk monitoring during active service, with regular review of behavioural signs, recovery, workload, and health-related safeguards. Finally, the sixth tier should support lifecycle review, including temporary withdrawal, workload reduction, retraining, or retirement when the dog’s welfare, health, or willingness to participate changes over time. Taken together, this framework provides a practical way to organise preparation as a welfare-centred, context-sensitive, and career-long process. Implementation of the lifecycle framework could be proportionate to setting risk, with higher-risk environments requiring stricter hygiene controls, site-specific preparation, and more frequent welfare review [1,15,16,19,20]. The framework does not claim that each individual tier is novel. Its contribution is integrative: it brings established welfare, dyadic, governance, risk-management, and lifecycle principles into a single organising structure that can be operationalised, tested, and refined empirically.

A further barrier to progress is inconsistent reporting. Many studies omit essential dog-level information such as age, breed, sex, neuter status, health status, selection criteria, training history, handler experience, session frequency, and welfare monitoring, which limits comparison and replication [5,66]. Better reporting would improve the ability to identify which preparation variables are associated with canine welfare, human outcomes, retention, and safety and would give certifying bodies a stronger evidence base for revision of standards [6,66]. A structured set of recommended minimum reporting standards for studies on dog-assisted interventions is presented in Table S1 in the Supplementary Materials (Recommended minimum reporting standards for studies on dog-assisted interventions).

The next research step should move beyond asking whether dog-assisted interventions help humans and instead examine how therapy dogs can be selected, prepared, supported, monitored, and retired in ways that protect welfare and sustain programme quality [7,13,15,49]. This requires collaboration across veterinary science, behaviour, welfare science, epidemiology, psychology, healthcare, education, and policy, with canine welfare treated as a primary outcome rather than a secondary consideration [14,19] (see Table 4 for details). Priority areas include comparative governance evaluation, validation of selection tools, handler competency assessment, multimodal welfare datasets, and longitudinal evidence on workload, cumulative strain, and retirement timing [6,9,23,43,45,46,47,48,50,51,53,54,63,64,65,66].

For programmes and institutions, the practical implication is that therapy dog welfare should be built into organisational design rather than left to individual goodwill. Written welfare policies should define acceptable preparation methods, handler responsibilities, visit exclusion criteria, monitoring procedures, and retirement pathways; assessment should address both the dog and the human–dog team; handler training should cover stress signals, positive welfare, agency, hygiene, environmental management, and ethical decision making; and dogs should receive setting-specific preparation before deployment [1,6,15,17,24,48,50,51]. Host sites should also adopt written policies on infection control, participant eligibility, hand hygiene, contact rules, allergies, incident reporting, rest areas, and staff responsibility, while programmes should monitor subtle changes in motivation, recovery, health, and behaviour over time and normalise retirement as a planned welfare-positive transition rather than a sign of failure [14,16,49,56].

Overall, the field has reached a point where expansion of animal-assisted interventions should be matched by stronger standards for the animals involved [1,6,9,34]. Harmonisation does not require identical laws in every country; it requires agreement on core principles: therapy dogs should be individually suitable, positively prepared, competently handled, contextually assessed, actively monitored, protected from preventable health risks, and supported across their working lifespan [15,16,19,20]. In that sense, therapy dog preparation should be understood as a career-long process of welfare assurance rather than a one-time certification event.

### 4.2. Limitations

This review has several limitations. It is a narrative synthesis rather than a systematic review or meta-analysis, so the findings necessarily reflect interpretive judgement as well as evidence. The search strategy was also not terminologically exhaustive and did not include several contemporary or setting-specific expressions, including “animal-assisted service”, “dog-assisted service”, “canine-assisted education”, “school dog”, “wellbeing dog”, and “well-being dog”. Although broader overlapping terms and supplementary citation-searching methods were used, this omission may have reduced retrieval of publications indexed exclusively under these labels, particularly within educational, school-based, and wellbeing contexts. The findings should therefore be interpreted as a structured narrative synthesis rather than an exhaustive account of all terminology and service contexts represented in the field. The governance comparison also depends on publicly available documents, meaning that internal procedures may not be visible. Moreover, although the overall search strategy was limited to English-language publications or publications with English-language abstracts, the grey literature search was conducted in English and did not involve a systematic country-by-country search of all European Union Member States or all countries geographically located in Europe. These language and geographical restrictions may have limited the identification of national legislation, ministerial guidance, organisational standards, and certification procedures published exclusively in national languages. Consequently, the European frameworks identified in this review should be interpreted as illustrative rather than comprehensive. Searches conducted in individual national languages may identify additional governance documents and could therefore lead to different conclusions regarding the availability and distribution of therapy-dog governance arrangements across Europe. In addition, the review focuses on Europe and the United States, so transferability to other regions should be made cautiously. Some relevant evidence also comes from adjacent fields such as companion-dog training, assistance-dog selection, veterinary behaviour, and general animal welfare science, which are highly informative but not perfectly equivalent to therapy dog work [9,23,45,46,47,64].

## 5. Conclusions

Therapy dog preparation for animal-assisted interventions remains unevenly governed despite the growing involvement of dogs across healthcare, education, social care, and community settings. This review shows that preparation cannot be reduced to obedience, sociability, or a single certification event but instead requires integrated attention to selection, health screening, context-suitability assessment, reward-based training, handler competency, welfare monitoring, health and zoonotic risk management, workload regulation, and retirement planning [15,16,19,20].

A central conclusion is that readiness should be treated as a dynamic property of the human–dog team within a specific intervention context rather than as a fixed attribute of the dog alone [6,15]. The proposed tiered lifecycle framework begins with foundational health and behavioural screening, moves through context-specific suitability assessment, reward-based preparation, handler competency, and active-service welfare monitoring, and ends with workload review, temporary withdrawal when needed, and planned retirement. In this way, the framework provides a practical structure for aligning therapy dog governance with animal welfare, team competence, and the demands of the intervention setting.

Advancing the field now depends on stronger longitudinal welfare research, validated selection and handler competency tools, more transparent reporting of preparation variables, and comparative evaluation of governance models. Without these developments, therapy dog programmes will continue to expand faster than the evidence and systems needed to safeguard canine welfare, human safety, and intervention quality.

## Figures and Tables

**Table 1:** Search strategy and source documentation for the narrative review.

Source	Search Strategy/Retrieval Method	Search Date	Records Retrieved	Records Screened	Records Retained for Synthesis	Purpose
PubMed	Core search string combining therapy dog/dog-assisted intervention terms with preparation, welfare, handler, zoonotic, and governance terms	Feb–Jun 2026	420	51	28	Biomedical, veterinary, public health, and zoonotic risk literature
Scopus	Core search string adapted to title, abstract, and keyword fields	Feb–Jun 2026	510	120	34	Interdisciplinary AAI, animal behaviour, psychology, education, and welfare literature
Web of Science	Core search string adapted to topic search	Feb–Jun 2026	330	82	21	Citation-indexed literature across veterinary, behavioural, social, and health sciences
Google Scholar	Targeted searches using topic-specific terms; first 100 results per search screened unless relevance saturation occurred earlier	Feb–Jun 2026	199	60	18	Supplementary literature, recent publications, and citation chasing
Organisational and governmental websites	Targeted retrieval from IAHAIO, ISAAT, Pet Partners, Therapy Dogs International, Alliance of Therapy Dogs, American Kennel Club, Pets As Therapy, Italian NRC AAI, Austrian ministry sources, AVMA, and EU Human Dog Team project materials	Feb–Jun 2026	Not applicable	38	18	Certification standards, governance documents, policy frameworks, and professional guidance
Backward and forward citation searching	Reference lists of key reviews and highly relevant empirical studies were screened; forward citations checked using Google Scholar and database citation tools	Feb–Jun 2026	38	38	20	Identification of seminal, missed, or recently citing literature

Source: Authors’ own work.

**Table 2:** Comparative overview of selected therapy dog governance, certification, and standards frameworks in Europe and the United States.

Framework/Jurisdiction	Status	Dog or Team Assessment	Handler Preparation	Welfare Monitoring	Health/Zoonotic Risk Provisions	Re-Evaluation or Continuing Oversight
IAHAIO White Paper	International non-binding guidance	Provides principles rather than direct certification	Provides definitions and broad practice guidance	Emphasises animal wellness, One Health, and One Welfare	Addresses animal wellness and responsible practice at principle level	Recommends responsible oversight but does not certify teams
ISAAT	International accreditation and standards body	Accredits curricula and training frameworks rather than directly regulating all teams	Accredits continuing education and human–dog AAI team training	Emphasises dignity, respect, ethical practice, and animal welfare	Addressed through standards and accredited curricula	Continuing oversight depends on accredited programme requirements
Animal Assisted Services International (AASI)	International non-profit membership, standards, competencies, and accreditation organisation.	Addresses therapy animals and assessment as standards domains	Includes handler standards and competency expectations	Includes animal welfare as a distinct standards domain	Includes risk management as a distinct standards domain	Functions as guidance unless adopted by organisations or institutions
Pet Partners, USA	Voluntary certification/registration organisation	Requires team evaluation, including roleplay-based assessment	Requires handler education and written assessment	Public materials emphasise handler responsibility to advocate for the animal and end or modify interactions when needed	Includes safety and best-practice guidance	Public programme standards indicate team evaluation every two years
Therapy Dogs International, USA	Voluntary registration organisation	Evaluates dog–handler teams; evaluation begins with observation of the team	Public materials state therapy dog classes are not required	Welfare provisions are not fully specified in public testing summary	Health and safety requirements exist at programme level but vary in detail across public documents	Re-evaluation expectations are organisation-specific
AKC therapy dog title programme, USA	Recognition/title programme, not standalone certification	Recognises visits performed through recognised therapy dog organisations	Does not itself function as primary handler-training system	Not a welfare-monitoring system	Not a health risk governance system	Title recognition is distinct from team recertification
Italian National Guidelines/NRC AAI	Nationally coordinated guideline and institutional framework	Includes attention to health, aptitude, professional roles, and multidisciplinary team structure	Specifies professional roles and training requirements within AAI teams	Incorporates veterinary and welfare oversight within a One Health-oriented model	Promotes standardised operating protocols and collaboration between human and veterinary medicine	Oversight depends on national guideline implementation and institutional practice
Austria therapy companion dog framework	Formal national legal/ministerial framework	Requires training of dog and handler plus positive expert assessment	Handler training is part of eligibility for the designation	Legal and assessment materials include quality assurance, animal welfare, rest, health maintenance, and regular health checks	Includes health suitability, health maintenance, and quality-assurance expectations	Assessment and quality-assurance criteria are specified through ministerial guidance
Human Dog Team project, Europe	Erasmus+ project-based guidance and curriculum	Provides handbook and training materials for human–dog teams	Includes curriculum content for handlers and teams	Addresses selection, training, behaviour, veterinary basics, and health and safety	Includes training content on canine health and safety during work	Does not constitute a permanent statutory certification system
Pets As Therapy, UK	Charity-led voluntary assessment model	Assesses handler and pet as a team through temperament assessment	Provides registration and volunteer guidance	Public assessment materials acknowledge that visits may be tiring and stressful for pets	Requirements are managed through organisational and site-level policies	Reassessment and continuing oversight are organisation-specific

Note: The frameworks were identified through a targeted English-language grey-literature search and are presented as illustrative examples rather than as a comprehensive or representative mapping of governance in either region. The absence of a country, jurisdiction, organisation, or framework should not be interpreted as evidence that relevant governance procedures do not exist. Source: Authors’ synthesis based on the publicly available framework documents cited in the text.

**Table 3:** Summary of evidence synthesis across core therapy dog preparation domains.

Preparation Domain	Main Synthesis Finding	Current Strength of Evidence	Key Limitation	Implication for Standards
Candidate selection	Suitability depends on individual temperament, health, sociability, resilience, recovery capacity, and context-specific demands	Moderate	No universally validated therapy dog selection tool	Use multi-stage screening, individual assessment, health checks, and context-specific suitability evaluation
Training methodology	Reward-based, gradual, and consent-oriented preparation is most consistent with welfare science	Moderate, partly extrapolated from companion-dog training literature	Limited therapy-dog-specific training method studies	Certification should assess training history and methods, not only behavioural outcomes
Handler competency	Handler knowledge, stress recognition, environmental management, and decision making are central to welfare and safety	Moderate but fragmented	Lack of validated handler competency tools	Assess the human–dog team and require handler education, observation, and reassessment
Welfare monitoring	Welfare outcomes vary by dog, handler, setting, workload, and recovery; multimodal monitoring is needed	Moderate	Small samples, inconsistent measures, limited longitudinal data	Combine behavioural, physiological, positive-welfare, and recovery indicators
Health and zoonotic risk management	AAI safety requires integrated veterinary screening, hygiene, visit exclusion rules, and incident reporting	Moderate	Inconsistent incorporation into certification systems	Treat health risk as a One Health preparation domain, not merely a site-level policy issue
Career lifecycle management	Workload, rest, temporary withdrawal, reduced duties, and retirement are under-standardised	Weak to emerging	Few longitudinal or whole-career studies	Develop lifecycle plans, workload logs, periodic welfare reviews, and retirement criteria
Governance	National, voluntary, international, and project-based systems vary substantially in scope and enforceability	Moderate descriptive evidence	Little comparative outcome evaluation	Compare governance models empirically and harmonise minimum welfare and handler standards

Source: Authors’ synthesis of the reviewed literature.

**Table 4:** Priority research agenda for therapy dog preparation and governance.

Priority Area	Recommended Study Design	Key Outcomes	Practical Value
Governance comparison	Cross-national comparative policy study	Welfare standards, handler requirements, incident reporting, recertification, attrition	Identifies which governance models are associated with stronger welfare safeguards
Candidate selection	Prospective cohort study following candidates from screening to deployment	Certification success, welfare outcomes, retention, incident rates	Validates selection tools and reduces inappropriate placement
Training methodology	Longitudinal observational study comparing reward-based, mixed, and aversive histories	Stress signals, recovery, handler responsiveness, withdrawal from work	Clarifies whether preparation methods affect therapy dog welfare and safety
Handler competency	Tool development and validation study	Stress recognition accuracy, decision making, environmental management, advocacy behaviour	Produces measurable standards for handler education and assessment
Dyadic functioning	Human–dog team compatibility study	Interaction quality, dog engagement, client outcomes, welfare indicators	Supports certification of the team rather than the dog alone
Positive welfare	Multimodal welfare study	Voluntary approach, play, affiliative behaviour, oxytocin/vasopressin where appropriate, recovery	Moves welfare assessment beyond distress detection
Zoonotic risk	One Health implementation study	Hygiene compliance, illness exclusion, pathogen risk protocols, incident reporting	Strengthens safety in hospitals, schools, and elder care settings
Lifecycle management	Multi-year cohort study	Workload tolerance, cumulative stress, health decline, retirement timing	Establishes evidence-informed workload and retirement guidance
Reporting standards	Audit of published AAI trials	Dog characteristics, training history, welfare reporting, withdrawal criteria	Improves transparency and comparability across studies

Source: Authors’ own work.

## Data Availability

No new empirical data were created or analysed in this study. This narrative review synthesised information from previously published articles and publicly available organisational, professional, and policy documents, all of which are cited in the manuscript. Data sharing is therefore not applicable to this article.

## Supplementary Materials

The following supporting information can be downloaded at: https://www.mdpi.com/article/10.3390/ani16162557/s1, Table S1: Recommended minimum reporting standards for studies on dog-assisted interventions.

## Abbreviations

The following abbreviations are used in this manuscript:

AASs	Animal-assisted services
AAIs	Animal-assisted interventions
C-BARQ	Canine Behavioral Assessment and Research Questionnaire
CONSORT	Consolidated Standards of Reporting Trials
SPIRIT	Standard Protocol Items: Recommendations for Interventional Trials
