# Supplementary material for: Preparing Therapy Dogs for Animal-Assisted Services: Governance, Welfare-Centred Preparation, and a Lifecycle Research Agenda Across Selected European and United States Frameworks
Source: Animals (Basel). 2026 Aug 16;16(16):2557. doi: 10.3390/ani16162557 (PMC13508879; doi:10.3390/ani16162557)
Supplement: Supplementary file 1 [file animals-16-02557-s001.zip › animals-4439528-supplementary.pdf]

Table S1. Recommended minimum reporting standards for studies on dog-assisted interventions.

Reporting domain	Recommended information
Dog characteristics	Age, sex, breed or breed type, neuter status, source, health status, prior experience
Selection	Screening tools, temperament assessment, exclusion criteria, evaluator qualifications
Training	Duration, methods, reinforcement strategy, aversive-method policy, context-specific habituation
Handler	Training, experience, relationship to dog, competency assessment, supervision
Intervention setting	Site type, participant population, sensory demands, infection-control requirements
Session structure	Duration, frequency, number of clients, type of contact, rest opportunities
Welfare monitoring	Behavioural indicators, physiological measures if used, positive welfare indicators, recovery measures
Health and hygiene	Vaccination, parasite control, grooming, hand hygiene, visit exclusion rules
Withdrawal and retirement	Temporary withdrawal criteria, incident response, retirement policy

Source: Authors' synthesis of the reviewed literature and relevant reporting guidance.
